# COVID-19 was not associated or trigger disease activity in spondylarthritis patients: ReumaCoV-Brasil cross-sectional data

**DOI:** 10.1186/s42358-022-00268-x

**Published:** 2022-11-22

**Authors:** Claudia Diniz Lopes Marques, Sandra Lúcia Euzébio Ribeiro, Cleandro P. Albuquerque, Samia Araujo de Sousa Studart, Aline Ranzolin, Nicole Pamplona Bueno de Andrade, Andrea T. Dantas, Guilherme D. Mota, Gustavo G. Resende, Adriana O. Marinho, Danielle Angelieri, Danieli Andrade, Francinne M. Ribeiro, Felipe Omura, Nilzio A. Silva, Laurindo Rocha Junior, Danielle E. Brito, Diana C. Fernandino, Michel A. Yazbek, Mariana P. G. Souza, Antonio Carlos Ximenes, Ana Silvia S. Martins, Glaucio Ricardo W. Castro, Lívia C. Oliveira, Ana Beatriz S. B. Freitas, Adriana M. Kakehasi, Ana Paula M. Gomides, Edgard Torres Reis Neto, Gecilmara S. Pileggi, Gilda A. Ferreira, Licia Maria H. Mota, Ricardo M. Xavier, Marcelo de Medeiros Pinheiro, the ReumaCoV-Brasil Registry

**Affiliations:** 1 Hospital das Clínicas – Universidade Federal de Pernambuco, Recife, Brazil; 2 Universidade Federal do Amazonas, Manaus, Brazil; 3 Universidade de Brasília (HUB-UnB-EBSERH), Brasília, Brazil; 4 Hospital Geral de Fortaleza (HGF), Fortaleza, Brazil; 5 Hospital Getúlio Vargas, Recife, Brazil; 6 Hospital de Clínicas de Porto Alegre – Universidade Federal do Rio Grande do Sul, Porto Alegre, Brazil; 7 Universidade Federal de São Paulo, Rua Borges Lagoa, 913/ 51-53, Vila Clementino, São Paulo, SP CEP: 04038-034 Brazil; 8 Hospital das Clínicas, Universidade Federal de Minas Gerais, Belo Horizonte, Brazil; 9 Fundação Hospitalar do Acre (Fundhacre), Rio Branco, Brazil; 10 Hospital dos Servidores de São Paulo – IAMSPE, São Paulo, Brazil; 11 Hospital das Clínicas, Universidade de São Paulo, São Paulo, Brazil; 12 Hospital Universitário Pedro Ernesto, Universidade do Estado do Rio de Janeiro, Abraão, Brazil; 13 Clínica Omura Medicina Diagnóstica, São Paulo, Brazil; 14 Faculdade de Medicina da Universidade Federal de Goiás, Goiânia, Brazil; 15 Instituto de Medicina Integral Professor Fernando Figueira -IMIP, Recife, Brazil; 16 Universidade Federal da Paraíba, João Pessoa, Brazil; 17 Universidade Federal de Juiz de Fora, Juiz de Fora, Brazil; 18 Hospital de Clínicas da Universidade Estadual de Campinas- UNICAMP, Campinas, Brazil; 19 Santa Casa de Belo Horizonte, Belo Horizonte, Brazil; 20 CIP - Centro Internacional de Pesquisa, Goiânia, Brazil; 21 Hospital de Clínicas, Universidade Federal de Uberlândia, Uberlândia, Brazil; 22 Hospital Governador Celso Ramos – Santa Catarina, Florianópolis, Brazil; 23 Hospital Santa Casa de Misericórdia de Vitória, Vitória, Brazil; 24 Faculdade de Ciências da Saúde Paulo Prata- FACISB, Barretos, Brazil; 25 UniCEUB, Brasília, DF Brazil; 26 Programa de Pós-Graduação em Ciências Médicas, Faculdade de Medicina, Universidade de Brasília (PPGCM-FM-UnB), Brazil, Brasília, DF Brazil; 27 Hospital Universitário de Brasília (HUB-UnB-EBSERH), Brasília, DF Brazil

**Keywords:** COVID-19, Hospitalization, Spondyloarthritis, Psoriatic arthritis, SARS-CoV-2

## Abstract

**Objectives:**

To evaluate the disease activity before and after COVID-19 and risk factors associated with outcomes, including hospitalization, intensive care unit (ICU) admission, mechanical ventilation (MV) and death in patients with spondylarthritis (SpA).

**Methods:**

ReumaCoV Brazil is a multicenter prospective cohort of immune-mediated rheumatic diseases (IMRD) patients with COVID-19 (case group), compared to a control group of IMRD patients without COVID-19. SpA patients enrolled were grouped as axial SpA (axSpA), psoriatic arthritis (PsA) and enteropathic arthritis, according to usual classification criteria.

**Results:**

353 SpA patients were included, of whom 229 (64.9%) were axSpA, 118 (33.4%) PsA and 6 enteropathic arthritis (1.7%). No significant difference was observed in disease activity before the study inclusion comparing cases and controls, as well no worsening of disease activity after COVID-19. The risk factors associated with hospitalization were age over 60 years (OR = 3.71; 95% CI 1.62–8.47, *p* = 0.001); one or more comorbidities (OR = 2.28; 95% CI 1.02–5.08, *p* = 0.001) and leflunomide treatment (OR = 4.46; 95% CI 1.33–24.9, *p* = 0.008). Not having comorbidities (OR = 0.11; 95% CI 0.02–0.50, *p* = 0.001) played a protective role for hospitalization. In multivariate analysis, leflunomide treatment (OR = 8.69; CI = 95% 1.41–53.64; *p* = 0.023) was associated with hospitalization; teleconsultation (OR = 0.14; CI = 95% 0.03–0.71; *p* = 0.01) and no comorbidities (OR = 0.14; CI = 95% 0.02–0.76; *p* = 0.02) remained at final model as protective factor.

**Conclusions:**

Our results showed no association between pre-COVID disease activity or that SARS-CoV-2 infection could trigger disease activity in patients with SpA. Teleconsultation and no comorbidities were associated with a lower hospitalization risk. Leflunomide remained significantly associated with higher risk of hospitalization after multiple adjustments.

**Supplementary Information:**

The online version contains supplementary material available at 10.1186/s42358-022-00268-x.

## Introduction

The COVID-19 epidemic is undoubtedly one of the greatest challenges facing humanity in the modern era. In just over 1 year, hundreds of articles on the evolution of SARS-CoV-2 infection in patients with immune-mediated rheumatic diseases (IMRD) have been published, but still there are some uncertainties regarding risk factors for unfavorable outcomes and clinical management in different types of IMRD [1].

One question that remains is the association between rheumatic disease activity and increased risk of SARS-CoV-2 infection, as well as whether COVID-19 could trigger disease activity. Some studies associate moderate to severe disease activity with increased risk of infection in patients with IMRD, as well that infection could trigger disease activity [2–7], but this results are contradictory.

In general, patients with spondyloarthritis (SpA) have a lower risk of infection, when compared to rheumatoid arthritis and systemic lupus erythematosus, possibly due to the little or almost no use of oral corticosteroids, recognized as the greatest risk factor for infection in these patients [8, 9]. In addition, as axial SpA (axSpA) patients are younger and with lower frequency of comorbidities reducing the risk of infection. However, immunobiological may increase risk when compared to the use of synthetic conventional disease modifying anti rheumatic drugs (scDMARD) [10].

Another point that deserves attention is the possible protective role of HLA-B27 against COVID-19. Since that Brewerton and Schlosstein [11–13] reported a close relationship between ankylosing spondylitis and HLA-B27, many other aspects have been pointed out over time, especially related to some protective role regarding infections (occurrence and severity) [14–17].

Some studies have evaluated the COVID-19 outcomes in SpA patients [6, 18–20]. However, most of them have small sample and they were analyzed together with other IMRDs and not in a separately way. This approach could reduce some selection bias found in mixing diseases with different particularities, including pathophysiological mechanisms, age, concomitant medication, comorbidities, glucocorticosteroids and other immunosuppressive drugs. Therefore, the aim of this study was to evaluate the disease activity association with COVID-19, as well risk factors associated with COVID-19 moderate/ severe outcomes, such as hospitalization, intensive care unit (ICU) admission, mechanical ventilation (MV) and death in patients with SpA.

## Methods

This is a cross-sectional analysis of the ReumaCoV-Brasil Registry. Details of the registry design were described elsewhere recently [21, 22]. Briefly, the ReumaCoV-Brasil is a multicenter, observational, ongoing prospective cohort study carried out to monitor adult IMRD patients with COVID-19 diagnosis comparing to IMRD without COVID-19, using a convenience sample. The patients started to be enrolled on May 20th, 2020.

Eligible patients were selected based on the identification of a case by the researcher, through telephone contact, outpatient appointment or during hospitalization related to COVID-19. The inclusion criteria were: (1) age over 18, (2) COVID-19 diagnosis, based on clinical symptoms AND/ OR polymerase chain reaction for SARS-CoV-2, AND/ OR antibody against SARS-CoV-2 (IgM or IgG), based on the Brazilian Ministry of Health criteria [23], and (3) prior diagnosis of SpA, according to usual classification criteria [24, 25]. The exclusion criteria were other immunodeficiency diseases, past organ or bone marrow transplantation, neoplasms within the last 5 years, current chemotherapy, HIV diagnosis and thymus diseases.

This study was registered at the Brazilian Registry of Clinical Trials—REBEc (RBR-33YTQC). Also, it was approved by the National Research Ethics Commission (CONEP) [Approval number 3,955,206, on April 5th, 2020], and all the patients read and signed the informed consent form before inclusion.

### Outcomes

Using a nationwide sampling strategy, it is a two-phase study: (1) cross-sectional evaluation (inclusion) with information about previous or current symptoms of COVID-19 and clinical characteristics at the baseline, which can be performed by telephone call (preferred because the social distancing) or by a face-to-face visit, if possible; (2) prospective follow-up concerning the IMRD characteristics with two face-to-face visits, every 3 months (3-month and 6-month assessment), after viral infection. The primary outcomes were the specific SpA disease activity changes after COVID-19, at four time points: (1) At baseline; (2) within 4–6 weeks after the SARS-CoV-2 infection; (3) 3 months after the inclusion (± 15 days); (4) 6 months after inclusion (± 15 days). If the patient is unavailable to perform a face-to-face visit at baseline because social distancing, the physician may use the clinical data within the last 6 months (a period without any COVID-19 evidence).

The disease activity assessment was performed using a global physician assessment (GPA), using a numerical visual analog scale (VAS), ranging from 0 to 10 (being zero no activity and 10 great activity), as well specific and validated disease activity measurements. Pre-COVID data were obtained from the notes of the patient's medical record, in a consultation carried out in the last 6 months and the post-COVID data were obtained at the time of data collection for this study. For patients with axSpA, the disease activity measurement considered were BASDAI (Bath Ankylosing Spondylitis Disease Activity Index) [26], ASDAS-ESR (Ankylosing Spondylitis Disease Activity Score using erythrocyte sedimentation rate) [27] and ASDAS-CRP (Ankylosing Spondylitis Disease Activity Score using Protein C Reactive) [26] as well the frequency of isolated clinical manifestations before (cases and controls) and after COVID-19 (only for cases). For PsA patients, besides disease activity PGA, isolated clinical manifestations of disease activity were used, as well as minimal disease activity (MDA) [28] criteria.

Outcomes related to COVID-19 severity were assessed and classified according to the care needed for each patient. Mild COVID-19 required only ambulatory care, moderate COVID-19 required non-intensive hospital treatment, including emergency room and stay for more than 24 h; and severe COVID-19 required admission to an ICU, MV, or led to death. All participants included in this analysis had been prospectively monitored until complete endpoints resolution.

### Covariates

Demographic data such as age, sex, work situation and social distancing during the pandemic, as well diagnosis and treatment of IMRD, comorbidities (https://www.who.int/classifications/icd/icdonlineversions/en/), clinical characteristics, COVID-19 management and their endpoints, were collected using a Research Electronic Data Capture (REDCap) database (https://www.project-redcap.org/), through telephone call or face-to-face interview, if permitted by local health recommendations. In case of hospitalization, the data were collected directly with the patient, if possible, or from medical records. In cases where the death was notified, data were collected directly from a family member, who authorized the data inclusion.

### Statistical analysis

To characterize the patient profile, the frequencies percentage and mean and standard deviation (SD) of variables were calculated. Comparisons of means between two groups were performed using the Student's t test for independent samples. To verify normality data, it was applied the Kolmogorov–Smirnov test. In case normality violation, it was used the Mann–Whitney non-parametric test. The chi-square association test was used to assess the association among categorical variables with standardized adjusted residual calculation, or Fischer's exact test for small samples. The final logistic regression model used moderate/ severe forms as dependent variable and appropriate adjustments were performed considering all independent variables that had statistical significance up 10% in the univariate analysis. *p* value was set as significant if below 5%. Statistical analyzes were performed using the SPSS 20.0 statistical software.

## Results

### Demographic and clinical data

Results are reported in accordance with STROBE guidelines.

From May 20th, 2020, to June 30th, 2021, a total of 1984 IMRD patients were included: 1093 (55.1%) cases with COVID-19 and 891 (44.9%) controls without COVID-19. Considering only 353 (17.8%) SpA patients, 229 (64.9%) had axSpA, 118 (33.4%) with PsA and 6 (1.7%) had enteropathic arthritis (Additional file 1).

The mean age of the SpA sample was 48.4 years (12.4), with no significant differences compared to cases and controls. Most of them were active at work (52.4%) and 138 (39.1%) reported no social distancing during the pandemic. The main comorbidities were hypertension (36.0%) and diabetes (14.4%). Current smoking was just reported by 6.8% % of sample. PsA patients were older (53.4 vs. 45.6 years, *p* = 0.0001), with greater number of housewives (19.5% vs. 4.8%, *p* = 0.001), higher frequency of being inactive at work (55.6% vs. 42.5%, *p* = 0.022) and higher diabetes frequency (23.7% vs. 10.0%, *p* = 0.001), hypertension (45.8% vs. 31.0%, *p* = 0.007) and obesity (20.3% vs. 10.9%, *p* = 0.017). Axial SpA patients had a higher frequency of not having any comorbidities (43.2% vs. 28.0%, *p* = 0.006).

HLA-B27 status was available in 193 patients (54.6%) and the positivity was found in 120 (62.2%) of SpA patients (73.2% in axSpA and 20.0% in PsA, *p* = 0.000).

TNFi and methotrexate were the DMARDs more used by SpA patients (66.3% and 23.1%, respectively). PsA patients used more frequently IL-17 inhibitors, leflunomide and methotrexate than axSpA patients (22.9% vs. 10.0%, *p* = 0.001; 11.9% vs. 0.9%, *p* = 0.0001; 40.7% vs. 14.0%, *p* = 0.000, respectively). On the other hand, axSpA patients were taking more TNFi and sulfasalazine than PsA patients (74.2% vs. 50.8%, *p* = 0.0001, and 11.8% vs. 0.8%, *p* = 0.0001, respectively). There is no difference between the groups concerning oral corticosteroids (*p* = 0.167).

Comparing cases and controls, it was observed that in the case group the frequency of professions with public exposure was higher (*p* = 0.038), as well the frequency of lung disease (*p* = 0.018), greater weight (*p* = 0.006) and abdominal circumference (*p* = 0.018). The control group had lower frequency of comorbidities than the case group (*p* = 0.03). Table 1 summarizes the clinical and epidemiological data, comparing cases and controls in the complete sample, as well as in the axSpA and PsA groups. Data from patients with enteropathic arthritis were not included due to the small sample size (6 patients).

**Table 1:** Clinical and epidemiological data, comparing cases and controls in the complete spondyloarthritis sample, according to axial spondyloarthritis (axSpA) or psoriatic arthritis (PsA)

	Total (N = 353)					axSpA (n = 229)					PsA (N = 118))				
	Cases (n = 210)		Controls (n = 143)		*p*	Cases (n = 132)		Controls (n = 97)		*p*	Cases (n = 75)		Controls (n = 43)		*p*
	n	%	N	%		n	%	n	%		n	%	n	%	
Male	112	57.4	83	42.6	0.382	77	53.5	67	46.5	0.096	35	70.0	15	30,0	0.213
Profession															
Health care, security, and education	46	70.8	19	29.2	**0.038**	33	67.3	16	32.7	0.114	12	80.0	3	20.0	0.250^(a)^
Work status—active	117	63.2	68	36.8	0.109	80	61.1	51	38.9	0.200	37	71.2	15	28.8	0.113
No social distancing	81	58.7	57	41.3	0.835	51	56.7	39	43.3	0.846	30	63.8	17	36.2	0.960
HLA-B27 positivity^#^	69	57.5	51	42.5	0.704	64	57.1	48	42.0	0.514	5	62.5	3	37.5	0.677^(a)^
Comorbidities, N (%)															
Hypertension	81	63.8	46	36.2	0.218	44	62.0	27	38.0	0.374	35	64.8	19	35.2	0.795
Diabetes mellitus	32	62.7	19	37.3	0.609	12	52.2	11	47.8	0.576	20	71.4	8	28.6	0.322
Obesity	31	62.0	19	38.0	0.696	16	64.0	9	36.0	0.495	15	62.5	9	37.5	0.904
Cardiopathy	16	72.7	6	27.3	0.192	9	75.0	3	25.0	0.246^(a)^	7	70.0	3	30.0	0.745^(a)^
Lung disease	12	92.3	1	7.7	0.018	9	90.0	1	10.0	0.047^(a)^	3	100.0	0	0	–
Kidney disease	4	57.1	3	42.9	1.000	1	25.0	3	75.0	0.314^(a)^	3	100.0	0	0	–
Others	57	59.4	39	40.6	0.979	33	58.9	23	41.1	0.823	22	57.9	16	42.1	0.378
Number of comorbidities															
None	70	52.2	64	47.8	0.030	50	50.5	49	49.5	0.056	20	60.6	13	39.4	0.678
One or more	67	31.9	31	21.7	0.035	43	32.6	20	20.6	0.045	22	29.3	10	23.3	0.475
Current smoking	12	54.2	11	45.8	0.570	7	58.3	5	41.7	0.975	6	50.0	6	50.0	0.303
Oral corticosteroids	14	82.4	3	17.6	0.074	6	75.0	2	25.0	0.472^(a)^	8	100.0	0	0	0.027^(a)^
DMARDs															
TNF inhibitors	139	59.4	95	40.6	0.962	100	58.5	70	41.2	0.539	38	63.8	21	36.2	0.959
IL17 inhibitors	28	56.0	22	44.0	0.587	11	47.8	12	52.2	0.315	17	63.0	10	37.0	0.942
IL12/23 inhibitors	1	33.3	2	66.7	0.568	0	0	0	0	-	1	33.3	2	66.7	0.553^(a)^
Leflunomide	11	68.6	5	31.3	0.440	1	50.0	1	50.0	1.000	10	71.4	4	28.6	0.571^(a)^
Methotrexate	43	53.1	38	46.9	0.181	17	53.1	15	46.9	0.577	26	54.2	22	45.8	0.079
Sulfasalazine	19	65.5	10	34.5	0.490	17	63.0	10	37.0	0.551	1	100.0	0	0	-
Age in years, mean (SD)^(b)^	48.4 (12.9)		48.4 (11.6)		0.964	45.8 (12.7)		45.4 (10.7)		0.801	52.5 (12.4)		55.0 (11.3)		0.270
Ab. circumference (cm), mean (SD) ^(b)^	93.4 (15.3)		91.2 (15.2)		0.018	93.7 (14.8)		95.6 (15/3)		0.490	98.6 (13.0)		98.7 (6.0)		0.962
Weight (kg), mean (SD) ^(b)^	74.1 (6.5)		71.8 (6.1)		0.006	79.3 (16.8)		80.7 (16.7)		0.591	79.9 (4.1)		78.3 (16.7)		0.616
BMI (kg/m^2^), mean (SD) ^(b)^	28.7 (14.8)		28.0(9.4)		0.295	27.8 (5.1)		30.2 (18.3)		0.256	29.6 (5.3)		31.5 (18.2)		0.527
SBP (mmHg), mean (SD) ^(b)^	124.8 (17.5)		125.4 (5.2)		0.546	125.1 (14.7)		130.0 (18.5)		0.062	129.5 (16.1)		131.3 (17.3)		0.623
DBP (mmHg), mean (SD)^(b)^	79.4 (11.0)		79.6 (11.6)		0.757	80.0 (11.1)		81.2 (11.6)		0.482	82.5 (10.5)		81.8 (11.4)		0.755
Disease duration (years), mean (SD)^(b)^	10.4(9,9)		11.4 (9,1)		0.325	9.9 (10.1)		11.2 (8.8)		0.322	11.1 (9,6)		11.8 (9,9)		0.716

SD, standard deviation; Ab, abdominal; BMI, body mass index; SBP, systolic blood pressure; DBP, diastolic blood pressu

^#^Calculated only for patients who had the exam available; *p*—descriptive level of the chi-square test, Fisher's exact test (a), Student's t (b) or Mann–Whitney test (c)

## COVID-19 outcomes

The mean duration of COVID-19 symptoms was 13.3 (9.3) days, with no difference between PsA and axSpA (*p* = 0.412). The main frequent symptoms were headache (60.4%), myalgia (51.7%), anosmia (51.2%), dysgeusia (51.2%) and fever (50.7%). Additionally, 3.9% of patients were asymptomatic and they were diagnosed as COVID-19 because of a positive COVID test performed when a patient had contact with a confirmed case of COVID-19 or for preoperative assessment. Also, there were no differences between the symptoms presented by patients with PsA and axSpA. Lab confirmation of COVID-19 was obtained in 80.1% of samples, especially RT-PCR (58.5%). Teleconsultation were reported by 31.8% of patients. Fifty-eight SpA patients (28%) required hospital care, of whom 28 were hospitalized (48.2%) and 10 required ICU admission (17.2%). The number of patients who required MV was significantly higher in patients with axSpA (*p* = 0.026). The length of hospital stay was similar in both groups (Table 2). Furthermore, there was no difference concerning the mortality rate between axSpA and PsA patients (3 patients in the AxSpa group and 1 patient in PsA group; *p* = 0.382).

**Table 2:** COVID-19 outcomes in Axial Spondyloarthritis (axSpA) and Psoriatic Arthritis (PsA) patients

	Total (N = 207) *	axSpA (N = 132)	PsA (N = 75)	*p*
Symptom duration (days), mean (SD)	13.3 (9.3)	9.9 (10.1)	11.1 (9.6)	0.412^c^
Contact with a confirmed case, n (%)	114 (55.0)	73 (55.0)	41 (54.7)	0.495
Where the contact occurred^1^				0.443
Home, n (%)	75(65.8)	45 (61.6)	30 (73.2)	
Work, n (%)	25 (21.9)	17 (23.3)	8 (19.5)	
Another place, n (%)	14 (12.3)	11 (15.1)	3 (7,5)	
DMARDs withdrawal	76 (36.7)	48 (36.4)	28 (37.3)	0.889
Symptoms				
Headache, n (%)	125 (60.4)	83 (62.9)	42 (56.0)	0.331
Anosmia, n (%)	106 (51.2)	63 (47.7)	43 (57.3)	0.184
Fever, n (%)	105 (50.7)	69 (52.3)	36 (48.0)	0.554
Dysgeusia, n (%)	106 (51.2)	67 (50.8)	39 (52.0)	0.864
Myalgia, n (%)	107 (51.7)	67 (50.8)	40 (53.3)	0.721
Asthenia, n (%)	101 (48.8)	69 (52.3)	32 (42.7)	0.184
Cough, n (%)	94 (45.5)	59 (44.7)	35 (46.7)	0.784
Coryza, n (%),	76 (36.7)	51 (38.6)	25 (33.3)	0.447
Shortness breath, n (%)	70 (33.8)	49 (37.1)	21 (28.0)	0.182
Diarrhea, n (%)	66 (31.9)	41 (31.1)	25 (33.3)	0.736
Arthralgias, n (%)	51 (24.6)	38 (28.8)	13 (17.3)	0.066
Dizziness, n (%)	38 (18.4)	27 (20.5)	11 (14.,7)	0.301
Nausea, n (%)	36 (17.4)	25 (18.9)	11 (14.7)	0.436
Vomiting, n (%)	12 (5.8)	10 (7.6)	2 (2.7)	0.218^a^
Skin changes, n (%)	6 (2.9)	3 (4.0)	3 (2.3)	0.670^a^
Asymptomatic (only positive lab test), n (%)	8 (3.9)	4 (3.0)	4 (5.3)	0.464^a^
Lab test for SARS-CoV-2^2^	165 (80.1)	106 (80.3)	59 (79.7)	0.921
RT-PCR, n (%)	121 (58.5)	83 (62.9)	38 (50.7)	0.087
Serology (IgM/ IgG), n (%)	24 (11.6)	13 (9,8)	11 (14.7)	0.298
Telemedicine appointment, n (%)	66 (31.8)	42 (32.0)	24 (32.0)	0.985
Hospital care	58 (28.0)	39 (29.5)	19 (25.3)	0.496
Hospitalization^3,4^, n (%)	28/58 (48.2)	17/39 (48.7)	10/19 (52.6)	0.729
Intensive care unit admission^3,4^, n (%)	10/58 (17.2)	7/39 (18.0)	3/19 (15.8)	1.000^b^
Mechanical ventilation^5^, n (%)	7/58 (26.1)	7/39 (18.0)	0/19 (0.0)	0.026^**a**^
Hospital lengths stay (days), mean (SD)	13 (12.1)	13.0 (9.0)	13.8 (10.1)	0.626^b^

*p*—descriptive level of the chi-square test, Fisher's exact test (a), Student's t (b) or Mann–Whitney test (c); SD: standard deviation

^1^Only for cases with contact

^2^Only for patients with laboratory confirmed diagnosis

^3^Only for patients with hospital care

^4^Only for patients with AxSpA and PsA; 1 patient among the two with enteropathic arthritis was hospitalized

^5^Only for hospitalized or intensive unit care admission

*3 entheropatic arthritis not described

**Fig. 1:**
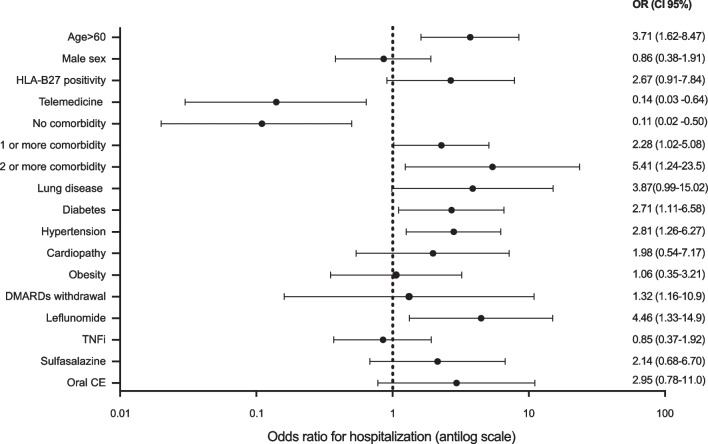
Forrest plot from the bivariate regression model for hospitalization in spondyloarthritis patients with COVID-19

Considering the bivariate regression model, the risk factors associated with hospitalization were age over 60 years (OR = 3.71; 95% CI 1.62–8.47, *p* = 0.001); one or more comorbidities (OR = 2.28; 95% CI 1.02–5.08); leflunomide treatment (OR = 4.46; 95% CI 1.33–24.9, *p* = 0.008). On the other hand, not having comorbidities (OR = 0.11; 95% CI 0.02–0.50, *p* = 0.001) played a protective role for hospitalization. Regarding the HLA-B27 positivity, it was not associated with hospitalization (Fig. 1).

After multiple adjustments, the risk factors associated with hospitalization in SpA patients were to be on leflunomide (OR = 8.69; CI = 95% 1.41–53.64; *p* = 0.023). Nevertheless, patients had had any telemedicine appointment during the COVID-19 (OR = 0.14; CI = 95% 0.03–0.71; *p* = 0.01) and no comorbidities (OR = 0.14; CI = 95% 0.02–0.76; *p* = 0.02) remained at final model as a protector factor.

Analyzing the specific effect of TNFi in SpA patients, including each group separately or in combination, no significant association was observed [PsA: OR = 0.38 (95% CI 0.09–1.56), *p* = 0.168) and axSpA: OR = 1.67 (95% CI 0.46–6.04), *p* = 0.426)].

As the frequency of ICU admission, MV and death was low, it was not possible to perform univariate or multivariate regression analysis.

### Disease activity before and after COVID-19

The mean time interval between the onset of symptoms and the first rheumatic assessment was 10 (9.7) weeks, with no difference comparing axSpA and PsA patients (10.2 vs. 9.0; *p* = 0.420).

### Axial spondylarthritis

**Fig. 2:**
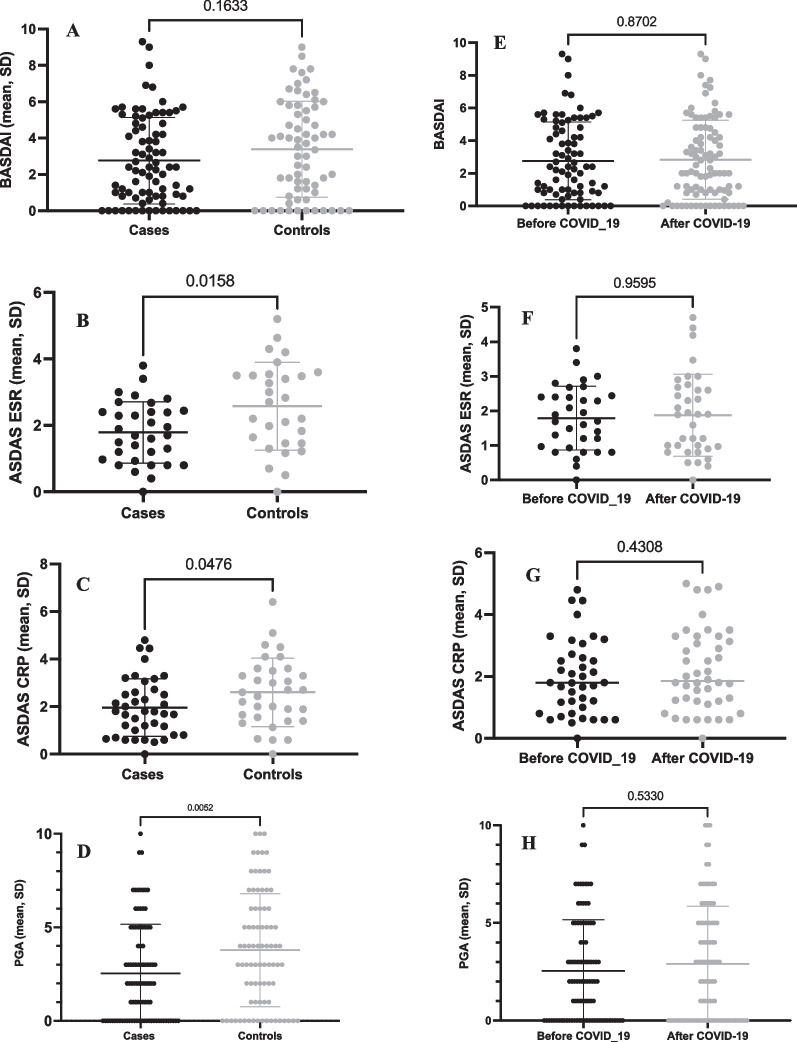
Disease activity in axial spondyloarthritis patients comparing cases and controls, and before and after COVID-19 in those with COVID-19, according to the BASDAI (**A**, **E**), ASDAS-ESR (**B**, **F**), ASDAS-CRP (**C**, **G**) and according to the physician global assessment (PGA)

Regarding the disease activity scores before COVID-19, it was observed that controls had significantly higher ASDAS-ESR and ASDAS-CRP, but not BASDAI (Fig. 2A–C). Comparing these three scores before and after COVID-19 in the case group, no statistically significant difference was observed for any one of them (Fig. 2E–G). The disease activity PGA pre-COVID showed a similar result, being higher in the control group (Fig. 2D), as well as there was no worsening after COVID-19 (Fig. 2H).

The frequency of active inflammatory back pain was higher in the control group (50.5% vs. 32.6%; *p* = 0.006) before the COVID-19. On the other hand, there was no significant difference regarding peripheral arthritis, enthesitis or uveitis between cases and controls (Table 3).

**Table 3:** Clinical manifestations of disease activity in patients with axial spondylarthritis and psoriatic arthritis before COVID-19

	Axial spondylarthritis					Psoriatic arthritis				
	Cases (n = 132)		Controls (n = 97)		*p*	Cases (n = 75)		Controls (n = 43)		*p*
	n	%	n	%		n	%	n	%	
No disease activity	45	34.1	27	27.8	0.314	23	30.7	14	32.6	0.831
Peripheral arthritis	13	9.8	10	10.3	0.909	17	22.7	12	27.9	0.525
Inflammatory back pain	43	32.6	49	50.5	0.006	6	8.0	3	7.0	0.840
Enthesitis	13	9.8	11	11.3	0.716	5	6.7	1	2.3	0.414
Uveitis	3	2.3	4	4.1	0.421	–	–	–	–	–
Dactylitis	–	–	–	–	–	6	8.0	1	2.3	0.209
Psoriasis	–	–	–	–	–	23	30.7	10	23.3	0.388
MDA	–	–	–	–	–	33	54.1	17	47.2	0.513

MDA, minimal disease activity

### Psoriatic arthritis

**Fig. 3:**
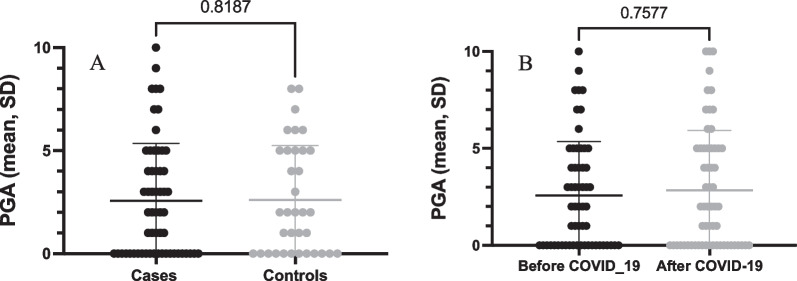
Disease activity in psoriatic arthritis patients comparing cases and controls (**A**) and before and after COVID-19 in those with COVID-19 (**B**), according to the physician global assessment (PGA)

Comparing cases and controls, no statistically significant difference was observed regarding pre-COVID disease activity in PsA patients (Fig. 3A), according to PGA. Considering the PGA before and after COVID-19, no worsening of disease activity was observed in the cases (Fig. 3B). The frequency of patients with active clinical manifestations was also similar in both groups (Table 3).

## Discussion

Our results showed no association between pre-COVID disease activity or that SARS-CoV-2 infection could trigger disease activity in SpA patients. Furthermore, teleconsultation and no comorbidities were associated with a lower hospitalization risk. Age over 60 years, have one or more comorbidities, lung disease, diabetes, hypertension and being on leflunomide were associated with hospitalization in bivariate analysis, and only leflunomide treatment remained significantly associated with higher risk of hospitalization after multiple adjustments, this used almost entirely by PsA patients. Regarding the moderate/ severe COVID-19 outcomes, there was no difference when we compared axSpA and PsA patients, except for higher MV probability.

Some recent studies have reported contradictory results when evaluated the association between disease activity and COVID-19. Hassel et al. [29] recently published data from the German cohort emphasizing that moderate to high disease activity was identified as an independent risk factor for hospitalization. However, most of them had rheumatoid arthritis (RA) (48%). Regarding only SpA patients, there was lower hospitalization risk compared with RA (OR = 0.46; 95% CI 0.23–0.91), suggesting that disease activity was more related to RA than SpA patients. Data from the COVID-19 Global Rheumatology Alliance physician-reported registry showed no association between disease activity and hospitalization in IMRD patients [6]. Also, results from Swiss Clinical Quality Management cohort [7] did not find no disease activity increment of disease in patients with axSpA, RA and PsA after COVID-19 infection using BASDAI, Rheumatoid Arthritis Disease Activity Index-5 and PGA, respectively.

Compared to other published studies that evaluated different IMRD altogether, our hospitalization rate was lower than reported by other cohorts [6, 19, 30]. Gianfrancesco et al. [6], from Global Rheumatology Alliance (GRA) study group, observed that patients with rheumatoid arthritis (RA) and systemic lupus erythematosus (SLE) were more hospitalized than SpA (46% in GRA and 29.4% in ours). However, Cordtz et al. [31], from the Danish cohort, and Sanchez-Piedra et al. [19], from Spanish cohort, reported a similar proportion to our results. Several aspects need to be considered among these studies to justify different results, such as eligibility criteria, COVID-19 definition, attendance protocols, healthcare institutions and service availability. However, considering specifically the patients with SpA from all these studies, there was also a relevant and heterogeneous range of hospitalization rate.

An unprecedented finding was the leflunomide as a significant risk factor to COVID-19 hospitalization in PsA patients, regardless of age and comorbidities. Although leflunomide has been previously associated with increased risk of infection requiring hospitalization after adjustment for important confounders [32], it has provided some beneficials actions beyond rheumatic diseases [33], such as anti-viral properties both in vitro and in vivo, including patients with COVID-19 [34–39], through inhibitor action against the dihydroorotate dehydrogenase (DHODH). On the other hand, it can upregulate HBV replication [40] and to reactivate hepatitis B, being indicated lamivudine prophylaxis to avoid reactivation. This is a result that needs to be better explored in larger and longer cohorts. Either way, this finding needs to be addressed by international recommendations for rheumatologists weighing the leflunomide withdrawal and its long half-life in PsA patients during the pandemic, especially in those on remission or low disease activity.

Interestingly, we did not find significant association with two aspects reported in other recently published studies, including a potential protective role of TNFi [6, 22] and a negative effect of corticosteroids with more severe forms of COVID-19 in IMRD patients with COVID-19 [6, 22, 29]. Some aspects could be postulated to explain the lack of significant potential protective role in SpA patients, particularly associated with pathophysiological differences, such as Th17/ Th1 pathway balance, neutrophils dysfunction, ability to induce NETosis, cathelicidines and a synergic or antagonic combination related to the TNF and IFN response. Thus, our data bring up a new data concerning TNFi in SpA patients: Would not the TNF blockade be enough to protect them? Could other pathways be involved? What the IL-17 role and neutrophils or Treg/Th17 cell imbalance in the uncontrolled systemic inflammation related to severe COVID-19? Could TNFi modulate the comorbidities in RA patients, differently from SpA patients?

Regarding the chronic use of oral corticosteroids and more severe forms of COVID-19, it has been reported in IMRD patients considered altogether, regardless of each underlying disease itself [6, 22, 29]. Differently of others, we performed a specific analysis from SpA patients to try better understanding its peculiarities and we did not find this effect. However, it is important to note that frequency of use of corticosteroids was quite low in this scenario.

Previous evidence supporting the association between HLA-B27 and lower viral load and long-term non-progression in chronic viral infection [17, 18]. Some mechanisms are postulated, including complicated pathways of viral escape from immunodominant HLA-B27-restricted virus-specific CD8+ T-cell epitopes, CD8+ T-cell polyfunctionality and functional avidity, thymic selection of CD8+ T-cell precursors, specific T-cell receptor repertoires and clonotypes, efficient antigen processing, and evasion from regulatory T-cell-mediated suppression. Thus, HLA-B27 could confer a protective effect against COVID-19, and HLA-B27 positive SpA patients could have lower occurrence and a less severe course of COVID-19 than those HLA-B27 negative. In our study, there was no statistically significant association between the positivity of HLA-B27 and the COVID-19 outcomes. Although not significant, a possible protective effect was observed regarding lower needed for MV in HLA-B27 positive patients when compared to HLA-B27 negative (OR = 0.44; CI 95% = 0.21–0.93; *p* = 0.08, data not shown). Our data suggest this relationship should be further studied in larger samples from SpA patients.

In addition, we could not confirm the GRA findings related to sulfasalazine. Recently, Strangfeld et al. [41] evaluating 3,729 patients with rheumatic diseases showed higher odds of death in sulfasalazine users when compared to methotrexate monotherapy (OR = 3.6; 95% CI 1.66–7.78). This association has also been reported in patients with inflammatory bowel disease (adjusted OR = 3.1; 95% CI 1.3–7.7) and severe COVID-19 [42]. Surprisingly, these two studies showed a drug with a low immunosuppressive effect and a potential immune role against other RNA viruses could have a negative impact regarding the SARS-CoV-2 [43]. The GRA authors highlighted a causal interpretation between sulfasalazine and death related to COVID-19 should not be made and other confounding factors may be involved, especially higher proportion of ever smokers compared to non-users, regardless of chronic lung disease, and csDMARDs combination.

When we analyzed another relevant finding from our study, the highest number of MV in axSpA than PsA patients, we cannot find any risk factor to justify it, even considering the former were younger and with less comorbidities and after adjustments for biological therapy. Thus, more prospective studies are needed to elucidate these findings. Also, the frequency and withdrawal proportion of conventional DMARDs were quite similar between them, as well as access to telemedicine and hospital care. However, it is important to highlight that from 6 axSpA patients on MV, 5 were HLA-B27 negative.

During the COVID-19 epidemic, telemedicine was a fundamental tool, in the sense of serving as a method of medical assistance to patients with rheumatic diseases, due to the restrictive measures of social contact, still adopted in some countries, such as Brazil. Thus, continuity of care for patients with IMRDs could be guaranteed through a virtual approach, although it will never entirely to replace in-person consultations [44]. In the pre COVID-19 era, a systematic review of telemedicine for rheumatic patients found a high degree of feasibility and satisfaction for interactions for consultation, treatment, and monitoring of disease activity [45]. Specifically focus on COVID-19, some previous reports showed that telemedicine could be feasible on management of IMRD as systemic lupus erythematosus (SLE) [46] and rheumatoid arthritis (RA) [47], and most of patients consider a phone consultation to be useful, particularly among patients who had low disease activity [47]. This important strategy has been also used in another settings, as breast cancer screening and follow-up [48], showing to be a suitable alternative during COVID-19 pandemic. In our study, healthcare outpatient appointments using telemedicine were reported by 32.9% of patients and it was associated with lower hospitalization risk. Thus, our data provide a significant finding of how the telemedicine could minimize the hospitalization risk in SpA patients, including orientations about maintenance of DMARDs and to avoid them on its own, management of symptomatic cases, preservation of health mental etc. Another important thing from our results is associated with clinical practice of rheumatologist, the need for leaving work and not attending professional activities in person. Our data did not show any significant related to moderate/ severe COVID-19 in the adjusted model final.

To our best knowledge, this is the first analysis considering specifically SpA patients and their main particularities in a real-life setting during the pandemic. In addition, it is important to note that all our patients had confirmatory test positive for COVID-19, particularly RT-PCR in more than 75% of sample, an important strength of our work to define the diagnosis. Many studies used only clinical or epidemiological criteria or self-reported diagnosis by patients. In addition, we presented outcomes data and its severity until its complete resolution regarding COVID-19 and provided more homogeneous data from a nationwide database with rheumatologists trained for collecting clinical details in a more systematized and consistent way. However, our study has some limitations, such as cross-sectional design and inherent inclusion bias regarding more severe cases and inconsistence to establish a cause-effect relationship, as well as the HLA-B27 status was available in only 60% of sample.

## Conclusion

Therefore, our data showed no association between pre-COVID disease activity or that SARS-CoV-2 infection could trigger disease activity in patients with SpA. Teleconsultation and no comorbidities were associated with a lower hospitalization risk. Leflunomide remained significantly associated with higher risk of hospitalization after multiple adjustments.

## Supplementary Information

**Additional file 1.** Flowchart of patients with immune-mediated rheumatic diseases enrolled in the ReumaCoV-Brasil study and Spondyloarthritis analysis.

## Data Availability

All data and material were anonymized on the REDCap platform and are available for auditing if necessary.
