# Supplementary figures and images for: COVID-19 was not associated or trigger disease activity in spondylarthritis patients: ReumaCoV-Brasil cross-sectional data
Source: Adv Rheumatol. 2022 Nov 22;62(1):45. doi: 10.1186/s42358-022-00268-x (PMC9685130; doi:10.1186/s42358-022-00268-x)

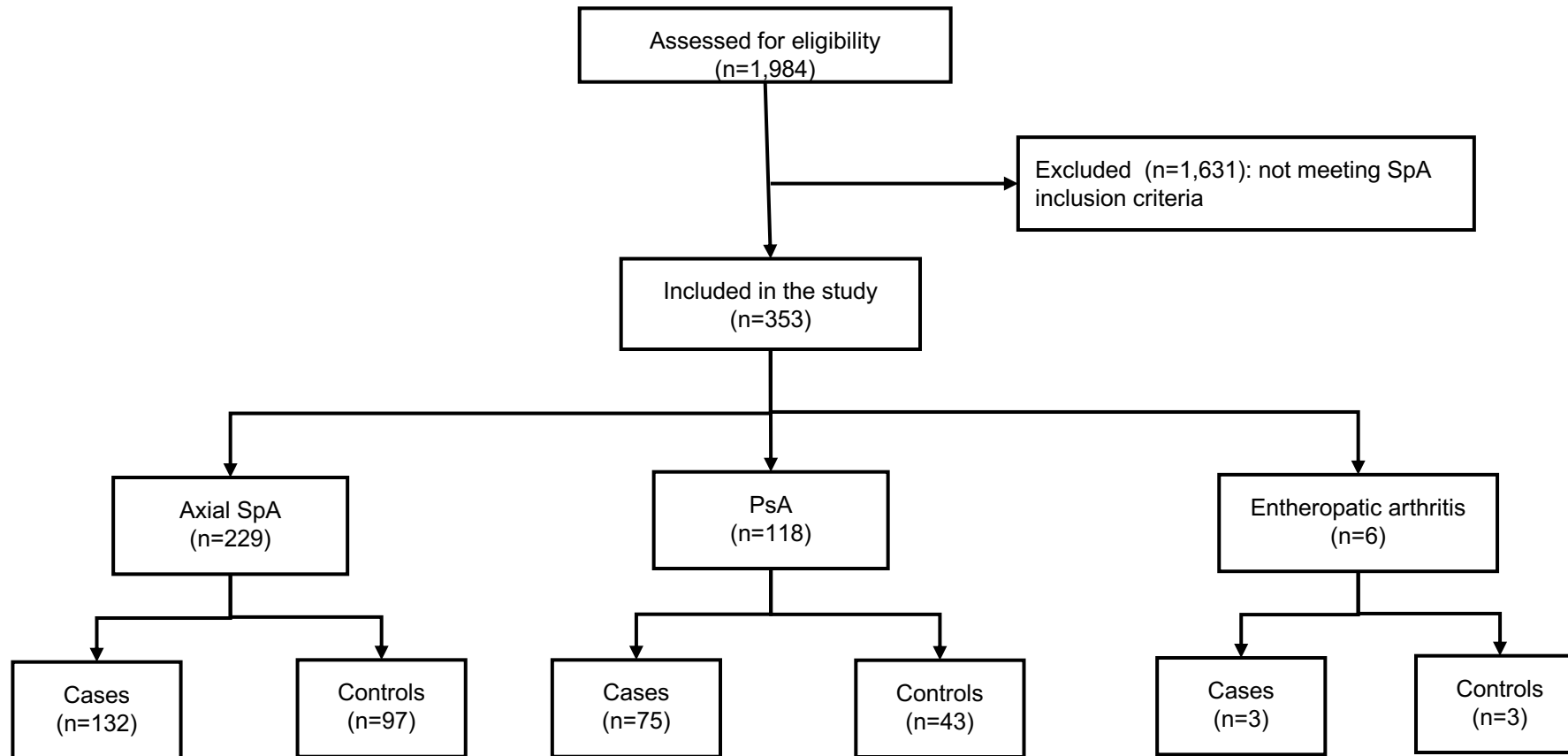

Supplement: Supplementary file 1 — Additional file 1. Flowchart of patients with immune-mediated rheumatic diseases enrolled in the ReumaCoV-Brasil study and Spondyloarthritis analysis. [file 42358_2022_268_MOESM1_ESM.pdf]
